# A Rare Case of Flare-Up of PID in Infertility Treatment

**DOI:** 10.1155/2015/146468

**Published:** 2015-10-27

**Authors:** Leena Wadhwa, Sanjana N. Wadhwa, Sunita Jindal

**Affiliations:** Department of Obstetrics and Gynecology, ESIC Post Graduate Institute of Medical Sciences and Research, Basaidarapur, Delhi 110015, India

## Abstract

*Case Presentation*. Mrs. X, 35 years old, case of primary infertility, was diagnosed to have genital tuberculosis on the basis of PCR positive and hysterolaparoscopy findings and received category I ATT for 6 months. Following ATT completion, her USG revealed no evidence of tuboovarian mass or hydrosalpinx. Since her tubes were patent, she underwent 3 cycles of ovulation induction and 2 cycles of IUI. The women presented with acute PID, five days after IUI, and was conservatively managed. She again presented 24 days after IUI with persistent low grade fever and abdominal pain. Suspecting relapse of genital tuberculosis, she was started on category II ATT. She had acute episodes of high grade fever with chills 2 weeks after starting ATT and MRI revealed bilateral TO masses suggestive of pyosalpinx. Emergency laparotomy was done, pus was drained, and cyst wall was removed and HPE was suggestive of chronic inflammation with few granulation tissues. ATT was continued for one year and the woman improved. *Conclusion*. The possibility of flare-up of PID (pelvic inflammatory disease) in treated case of tuberculosis undergoing infertility management should be kept in mind and aggressive management should be done.

## 1. Introduction

Pelvic inflammatory infection with intrauterine insemination is rare, probably in the range of 1 in 500 inseminations. Tuberculosis of the genital tract is a frequent cause of chronic pelvic inflammatory disease (PID) and infertility. When tuberculosis affects the genital organs of young females, it has the devastating effect of causing irreversible damage to the fallopian tubes, resulting in infertility that is difficult to cure both by medical and surgical methods [1]. Genital organs commonly involved include the fallopian tubes (95–100%), endometrium (50–60%), and ovaries (20–30%). The cervix (5–15%), vulva/vagina (1%), and the myometrium (2.5%) may also be involved [2, 3]. However, the disease often remains silent or may present itself with very few specific symptoms. As a result, the prevalence of genital tuberculosis (GTB) is most likely underestimated and may be found in 18% in African and Indian countries [1, 4]. Genital TB may be asymptomatic and diagnosis requires a high index of suspicion. Moreover, the disease may masquerade as other gynaecological conditions and can go unrecognised. Four-multiple-drug chemotherapy, comprising isoniazid, rifampicin, ethambutol, and pyrazinamide, is the mainstay of treatment.

Here we present the case of a young woman undergoing infertility treatment complicated with pelvic inflammatory disease (PID) after intrauterine insemination.

## 2. Case Presentation

Mrs. X, 35 years old, married for 6 years, case of primary infertility with hypothyroidism on levothyroxine 25 mcg, attended the infertility outpatient department of a tertiary care centre in May 2012. She had already undergone 2-3 cycles of ovulation induction. There was nothing significant in husband's history.

The patient was investigated for primary infertility.

Ultrasound on day 2 of menstrual cycle: uterus of normal size and endometrium thin, with antral follicle count of 3-4 follicles in each ovary and no hydrosalpinx or any adnexal mass.

Premenstrual endometrial biopsy: PCR/TB positive with AFB stain negative. HPE: secretory endometrium and no granulomas.

Hysterosalpingography: not done.

Hysteroscopy: cervical canal was normal. Bilateral ostia seen. Fibrosis in endometrium. Endometrial biopsy taken and sent for HPE.

Laparoscopy: uterus contour was normal. Tubercles were present all over. Biopsy was taken. In the left tube mild hydrosalpinx was present. Right tube was dilated. Both ovaries were stuck in POD. On chromopertubation with saline, bilateral spill was present.

Her endometrial biopsy report showed proliferative endometrium with no granuloma. TB PCR was negative.

Patient was started on category I antitubercular treatment in view of genital Koch's for six months. Treatment as per Revised National TB Control Program, in category I at the initial two months of therapy with INH (600 mg), rifampicin (450 mg), pyrazinamide (1500 mg), and ethambutol (1200 mg) in a thrice weekly schedule followed by INH (600 mg) and rifampicin (450 mg) in a thrice weekly schedule for the period of four months was given. After completion of her ATT course, her ultrasound showed uterus normal size with no evidence of tuboovarian mass or hydrosalpinx. Her antral follicle count was 3-4 follicles in each ovary.

The patient was planned for ovulation induction with intrauterine insemination. Before intrauterine insemination, there were no signs and symptoms of pelvic inflammatory disease. Pelvic examination was normal. She had three cycles of ovulation induction with two cycles of intrauterine insemination.

In her third cycle (August 2013), five days after intrauterine insemination the patient reported to the outpatient department with chief complaint of abdominal pain with fever. Acute pelvic inflammatory disease was diagnosed and the patient was admitted. Her laboratory investigations produced the following results: her blood cell counts were leukocytes of 12,000/*μ*L (4–11000) with neutrophilia, polymorph count of 86, hemoglobin of 10 g/dL (12–16), and platelets of 206,000/*μ*L (150–450,000). Her other serum parameters were within normal limits. Urine culture and blood culture were negative and ultrasound whole abdomen was within normal limits. She was started on intravenous antibiotics/antifungal. She became afebrile after 48 hours of intravenous antibiotics; TLC counts came down to 7700. She responded to treatment and was discharged.

24 days after intrauterine insemination (10 days after discharge from the hospital) the woman presented with low grade fever and abdominal pain. On her vaginal examination the uterus was of normal size. There was fullness and tenderness in pouch of Douglas. On ultrasound, the uterus was normal with endometrial thickness of 9.4 mm, right adnexa: 7 × 5.6 cm? tuboovarian mass. Left adnexa: hydrosalpinx of 7 cm adjacent to left ovary.

There was suspicion of relapse of tuberculosis.

The woman was started on ATT category II from DOTS (Directly Observed Treatment, Short-Course) centre. Treatment as per Revised National TB Control Program, in category II at the initial two months of therapy with streptomycin (750 mg) INH (600 mg), rifampicin (450 mg) pyrazinamide (1500 mg) ethambutol (1200 mg) in a thrice weekly schedule followed by INH (600 mg), rifampicin (450 mg), pyrazinamide (1500 mg), and ethambutol (1200 mg) in a thrice weekly schedule for period of one month followed by INH (600 mg), rifampicin (450 mg), and ethambutol (1200 mg) in a thrice weekly schedule for the period of five months was given. After 2 weeks of ATT the patient again presented with chief complaints of fever with chills and rigor and vomiting. On examination: per abdomen: 14-week palpable mass at the right side. On her vaginal examination uterus size was not made out. There was a firm mass with restricted mobility felt separately from the uterus. The patient was again admitted and started on intravenous antibiotics.

The patient continued to have fever and ultrasound showed persistence of tuboovarian masses with increasing TLC counts from thirteen thousands to twenty two thousands (13,000 to 22,000).

**Figure 1:**
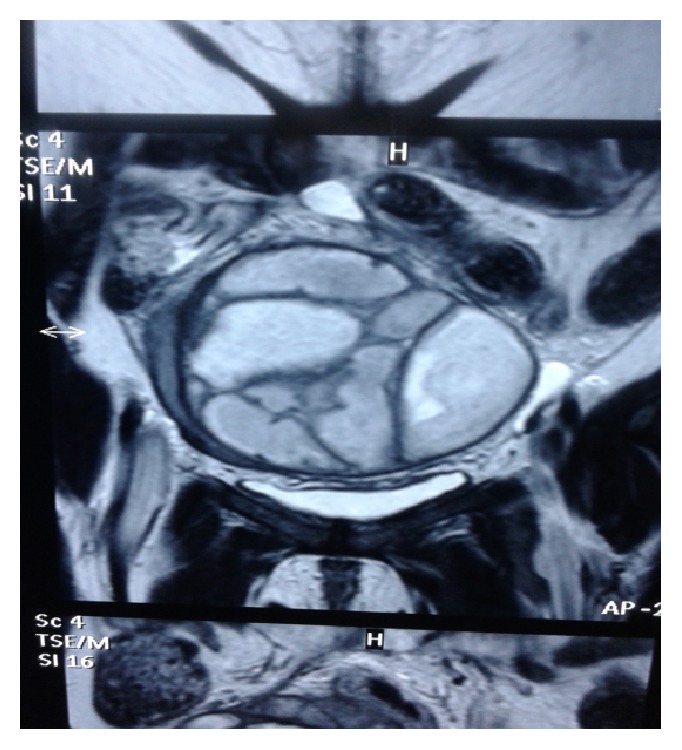
MRI of pelvis showing bilateral tuboovarian masses.

**Figure 2:**
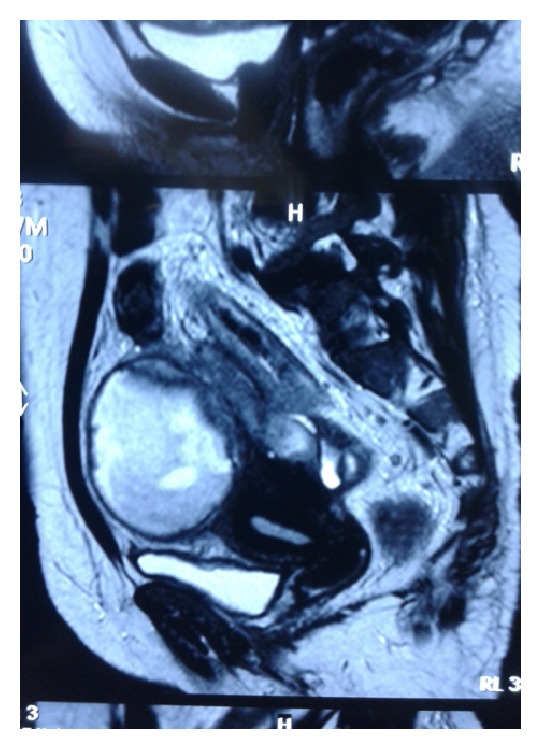
MRI of pelvis showing endometritis of uterus.

**Figure 3:**
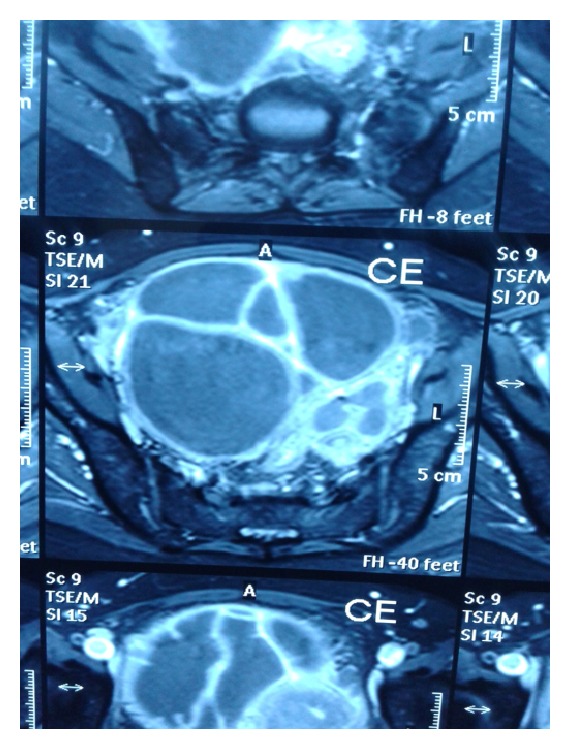
MRI of pelvis showing bilateral adnexal mass with septations.

MRI of pelvis showed bilateral tuboovarian masses 8 × 6 cm in right, 9 × 7 cm in left adnexa suggestive of pyosalpinx likely tubercular with septations with endometritis (Figures 1, 2, and 3).

The woman was taken up for* exploratory laparotomy* after four weeks of ATT. The intraoperative findings were kissing tuboovarian masses with a common cyst wall adherent to intestine and having loculated pus collection. 600 cc pus was drained and sent for AFB culture and Gram staining.

The woman continued on ATT category II and was discharged on same. The purulent discharge culture reported pus cells and Gram negative bacilli. HPE of cyst wall showed fibroconnective tissue with multiple microabscess consisting of neutrophils and macrophages, hemorrhages, and foci of chronic inflammation. Few granulation tissue areas were present.

**Figure 4:**
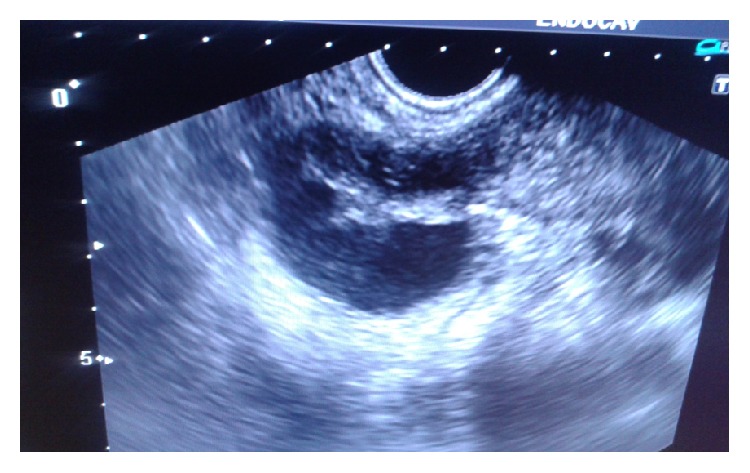
Ultrasound picture showing persistent tuboovarian masses.

The woman completed her ATT category II which she was given for one year. Now, she is doing well. Her ultrasound shows poor ovarian reserves with persistent tuboovarian masses (Figure 4). Hence, she is counselled for adoption/IVF with donor oocyte.

## 3. Discussion

Tuberculosis is one of the oldest known infections that has been difficult to eradicate. Genital tuberculosis (GTB) often remains silent or may present with very few specific symptoms. The tubercular bacilli can lie dormant in its latent form for years and can reactivate when host immunity is low. It has paucibacillary load which is not picked up with the presently available tests making diagnosis difficult. GTB is a pathology that mimics other diseases. Genital TB may present with a variety of gynaecological symptoms of infertility, menstrual disturbance, and chronic pelvic pain (Table 1) [5].

Although genital TB can occur in any age group, the majority of patients are in the reproductive age group, 75% being in the 20–45 years of age [6, 7].

Diagnosis is achieved most effectively through a combination of a high index of suspicion, especially in areas of low prevalence, thorough initial clinical assessment, and the use of appropriate investigations. High risk factors include a history of previous pulmonary TB infection, contact with a pulmonary TB sufferer, low socioeconomic background, drug abuse, HIV positive status, and history of chronic chest symptoms, night sweats, and weight loss.

Tuberculosis should be considered in a woman with high risk factors presenting with unexplained infertility, amenorrhea not explained by other causes, and pelvic infection that does not respond to ordinary treatment.

The diagnosis of GTB is a clinical challenge and is rarely achieved by consideration of clinical symptoms alone, given their low specificity. A proper diagnosis usually requires additional data derived from abdominopelvic ultrasonography, chest radiography, PPD skin tests, AFB staining, polymerase chain reaction analysis and/or histopathological evaluation, and specific cultures from intraoperative specimens, including invasive surgical procedures such as diagnostic laparoscopy. However, TB infection cannot be ruled out by negative results of these tests. Up to 92% of chest radiographs, over 90% of Ehrlich-Ziehl-Neelsen stains, and even pathological examinations can be negative in patients with extrapulmonary TB infection [8]. Although microbiologic culture is a gold standard for the definitive diagnosis of TB, a negative culture does not exclude the diagnosis of genital TB, as low microbiological burden, history of mycobacterial treatment or exposure to quinolone antimicrobial therapy, or inadequate culture preparation could cause a false-negative test [9].

Suspected tuberculous lesions in accessible sites such as the vagina, cervix, and the vulva may be biopsied directly. Endometrial tissue may be obtained by aspiration biopsy or by dilatation and curettage or directly at hysteroscopy. Endometrial biopsy is best performed in the premenstrual period. Menstrual fluid can be obtained from the vagina during the first day of menstruation for culture and microscopy. Less accessible lesions on the tubes, ovaries, and adnexae can be obtained at laparoscopy or laparotomy. Laparoscopic findings may include adhesions, tubal ovarian abnormalities, and ascites.

A negative premenstrual endometrial biopsy does not rule out genital TB as TB endometritis is seen in 50–60% cases only.

Only few case reports have reported flaring up of pelvic inflammatory disease after any surgical intervention.

There is a case report of ruptured tuboovarian abscess after intrauterine insemination. In this case a 27-year-old woman developed acute abdominal pain and fever one week after IUI. The diagnosis was PID. Intravenous antibiotics were started and she was still febrile after 3 days and had generalized tenderness on abdominal examination. Laparotomy was performed and left fallopian tube ruptured abscess was detected. Left salpingectomy was done [10].

There are few cases reported of postoperative flare-up of genital tuberculosis after total laparoscopic hysterectomy and vaginal hysterectomy [11].

Another case of flare-up of tuberculosis has been reported in a 28-year-old nulliparous woman with generalised miliary tuberculosis who underwent endometrial aspiration [12].

## 4. Conclusion

Before planning intrauterine insemination in infertile women especially with prior history of treated Koch's, one should rule out any signs and symptoms of PID. Reactivation of latent foci of tuberculosis can occur in immunocompromised state. The case should be thoroughly screened for pelvic infections. After IUI, if patient presents with symptoms of PID, one should be vigilant and aggressive management should be done.

## Figures and Tables

**Table 1:** Presenting symptoms of genital tuberculosis.

Symptom	Percentage of total
Infertility	43–74
Primary	55–78
Secondary	11–72
Normal menstrual function	50–88
Oligomenorrhoea	54
Amenorrhoea	14
Menorrhagia	19
Abdominal pain	42.5
Dyspareunia	5–12
Dysmenorrhoea	12–30

## References

[1] Varma T. R. (1991). Genital tuberculosis and subsequent fertility. *International Journal of Gynecology & Obstetrics*.

[2] Rajamaheswari N. Pelvic tuberculosis. http://www.Sunmed.org/pelvictb.html.

[3] Qureshi R. N., Samad S., Hamid R., Lakha S. F. (2001). Female genital tuberculosis revisited. *Journal of the Pakistan Medical Association*.

[4] Bapna N., Swarankar M., Kotia N. (2005). Genital tuberculosis and its consequences on subsequent fertility. *The Journal of Obstetrics and Gynecology of India*.

[5] Akbulut S., Arikanoglu Z., Basbug M. (2011). Tubercular tubo-ovarian cystic mass mimicking acute appendicitis: a case report. *Journal of Medical Case Reports*.

[6] Gatongi D. K., Gitau G., Kay V., Ngwenya S., Lafong C., Hasan A. (2005). Female genital tuberculosis. *The Obstetrician & Gynaecologist*.

[7] Nezar M., Goda H., El-Negery M., El-Saied M., Wahab A. A., Badawy A. M. (2009). Genital tract tuberculosis among infertile women: an old problem revisited. *Archives of Gynecology and Obstetrics*.

[8] Ilmer M., Bergauer F., Friese K., Mylonas I. (2009). Genital tuberculosis as the cause of tuboovarian abscess in an immunosuppressed patient. *Infectious Diseases in Obstetrics and Gynecology*.

[9] Klein T. A., Richmond J. A., Mishell D. R. (1976). Pelvic tuberculosis. *Obstetrics and Gynecology*.

[10] Moradan S. (2009). A ruptured tubo-ovarian abscess after intrauterine insemination; a case report. *Iranian Journal of Reproductive Medicine*.

[11] Singh N., Sharma A. K., Dadhwal V. (2008). Postoperative flare-up of genital tuberculosis: a clinical reality. *International Journal of Tuberculosis and Lung Disease*.

[12] Dadhwal V., Gupta N., Bahadur A., Mittal S. (2009). Flare-up of genital tuberculosis following endometrial aspiration in a patient of generalized miliary tuberculosis. *Archives of Gynecology and Obstetrics*.

